# A Period of Stress in Childhood

**Published:** 1910-11-15

**Authors:** V. A. Latham

**Affiliations:** Chicago


### A PERIOD OF STRESS IN CHILDHOOD.

BY V. A. LATHAM, M.D., D.D.S., F.R.M.S., CHICAGO.

Until recently this subject had not attracted the attention of medical or even dental writers to any extent, being only now and then touched on in connection with other matters. Lacking the aid of authority, I shall try to show its importance in daily practice and, at the same time, ask our members to look more attentively into their cases and see if the work of one of us is not worthy of more concentrated effort on our part earnestly to trace the subject better.

Who have better opportunities for observing the detrimental effect of a brain-cramming system of education at the expense of the bony and muscular systems than the wide-awake stomatologists.

"Why do my children's teeth decay so young?" is a common question. "I never had a tooth filled until I was twenty years old or more," says the questioner.

Look at the child with her, small, delicate frame with large head, bright eyes and a highly exalted nervous system. We are told that she is ready for high school at thirteen and began day school at four and a half; she does not care for sports, sits up till nine or ten, or later, with some neighbor children, and spends three to five hours a day practicing, which excites rather than soothes the nervous system. The amusements seem to be visiting other children, fudge parties, with late suppers, and later hours for retiring, and consequently too little sleep; dancing lessons on Saturday and going to the city for a matinee. It is one perpetual round of excitement, with no time for the daily care of the body, including the teeth, or assistance in the home duties, and far less desire to please others or be helpful to anyone but the child's immediate circle. The tendency of the times with the immense struggle to keep the family provided for the extremes in dress and fashions, the daily routine excitement with seldom a moment for relaxation, cannot help but ruin the finest heritage given to man, the vital structures by which his health, wealth, strength and happiness must be secured.

Prophylaxis—preventive medicine or hygiene—comes first and far above the mechanical art of dentistry, which is overdone. The crying demand of the nerves, however weak they are, to continue reacting by stimulation or excesses in food, drinks and automobiling, is fast developing a new set of disorders in vision, bones, muscles and heart strain, to say nothing of the nervous system. A recent recommendation of the use of the automobile (which to-day is seldom anything but a means for securing distance or speed instead of rest, tranquillity and change), in lung and heart diseases as a curative agent, should be carefully considered before it is accepted. The increase in neuralgia, or so-called neurotic pains, is well known and with the present ill-devised clothing it is no wonder that the speed and wind force should cause concussion and congestions with inflammatory conditions of the various parts of the body and especially the teeth. Again, the hurried meals at road-houses, excessive in many cases for the immediate needs, on account of the increased oxygen inhalation and faulty elimination, must cause auto-intoxication and many patients admit this when complaining of vague neuralgic pains or backache. The present urging of very young people into business positions of responsibility is a detriment to many; their hurry to escape the uncongenial atmosphere of the study rooms, and their almost universal carelessness in attending to their daily wants become a matter for thought. Austin Flint says: "The inexorable law of the survival of the fittest applies to man educated or uneducated, as well as to the lower animals, and it seems useless to educate man for work which he is physically unable to perform." The present rush to finish school work allows no extra time, as a rule, for broadening out in other lines, because time and money outweigh the study.

It should be remembered that the child consumes twice as much oxygen as the adult; and throws off a corresponding amount of carbonic acid; this is a gauge of its muscular activity and thus shows the need of the growing child for pure air in plenty. All muscular exertion is an expenditure of nervous force, hence, severe mental exertion and heavy physical strain should not be undertaken at the same time. Why should children not be forced to brain activity in tender years? Because, first, the brain grows most rapidly before the seventh year; secondly, the child is called on to assimilate and appropriate enough to nourish its rapidly growing brain and body, and at the same time to make good the wear and tear of its active nature. Is it not expecting too much of the digestive apparatus of the child to furnish material for its bodily development and, while so doing to supply food for an adult brain? Can we expect an overworked and excited child to digest its food properly and to furnish perfect material on which to feed its starving tissues? The food question of to-day is a real one in view of the extreme notions in children in regard to what they must eat, especially their repugnance to green vegetables.

Is it any wonder that the first seven years constitute a period of stress, with the brain growth, dentition, disorders incidental to infections and other childhood diseases? The rapid and great increase in the need for orthodontia, adenoid and nasal operations, the choreic cases, disorders of vision and digestion, etc., are evidence of this. Any mental strain shows itself on the teeth by increased caries as well as increased sensibility of the dentine, and especially as a cause for gingivitis and its allies, alveolitis and pyorrhea. Overwork of any vascular part such as the overtraining of the muscles for athletic contests, will give like results on the teeth. Mental shocks are also a serious menace to general and dental health. When brain activity is forced, a loss occurs to the system and it is called on to restore the requisite amount of phosphorus.[1] This is a vital component of both brain and bone in nearly equal amounts; hence, we must not forget that small doses of phosphorus produce good results in the reproduction of bone and are therefore a useful adjunct in so-called pyorrheal treatment. Care must be taken in its administration lest it become a detriment instead of a help, and the co-operation with laxatives is necessary.—*Journal A. M. A.*

